# In-person and online escape rooms for individual and team-based learning in health professions library instruction

**DOI:** 10.5195/jmla.2022.1463

**Published:** 2022-10-01

**Authors:** Rachel R. Helbing, Stefanie Lapka, Kathryn Richdale, Catherine L. Hatfield

**Affiliations:** 1 rrhelbin@central.uh.edu, Head of Health Sciences Libraries, Assistant Librarian, University Libraries, University of Houston, Houston, TX; 2 slapka@central.uh.edu, Health Sciences Librarian, Assistant Librarian, University Libraries, University of Houston, Houston, TX; 3 richdale@central.uh.edu, Associate Professor, College of Optometry, University of Houston, Houston, TX; 4 chatfiel@central.uh.edu, Director of Interprofessional Education & IPPEs, Clinical Professor, College of Pharmacy, University of Houston, Houston, TX

**Keywords:** Escape room, game-based learning, gamification, optometry, pharmacy, medicine, health professions students, teaching, instruction, active learning, team-based learning

## Abstract

**Background:**

A growing body of research demonstrates that adapting the popular entertainment activity “escape rooms” for educational purposes as an innovative teaching method can improve the learning experience. Escape rooms promote teamwork, encourage analytical thinking, and improve problem solving. Despite the increasing development and use of escape rooms in health sciences programs and academic libraries, there is little literature on the use of this method in health sciences libraries with health professions students.

**Case Presentation:**

Staff at a health sciences library collaborated with faculty to incorporate escape rooms into library instruction in a variety of settings (in-person, hybrid, online) and formats (team, individual) with health professions students from various disciplines (optometry, pharmacy, medicine). The escape rooms described in this paper offered unique experiences for students through active learning.

**Discussion:**

Important considerations when planning escape rooms for health sciences library instruction include deciding on team-based or individual design, calculating potential costs in time and money, deciding on an in-person, hybrid, or online format, and determining whether grades should be assigned. Escape rooms can be an effective strategy for library instruction in the health sciences, working in multiple formats to bring game-based learning to a variety of health professions students.

## BACKGROUND

The Health Sciences Libraries (HSL) at the University of Houston support seven health professions programs with three full-time librarians and three full-time library personnel across two locations. Health professions education requires students to consult the literature regularly; therefore, it is vital that students know how to locate and utilize appropriate resources.

Historically, students have been introduced to these skills for the first time as part of program-specific orientations or single lectures in select courses. These sessions can often be ineffective due to the challenges of lack of time in the curriculum, a student body with varying skills and experiences, and students' pre-existing perceptions of libraries. Additionally, most library material is delivered in a lecture format and does not allow time for application, lessening the likelihood for students to recall the information later. Library staff recognized it was time to rethink what students should gain from these sessions and redesign them to ensure students not only receive the knowledge they need but are able to independently apply the skills to access and search appropriate library resources.

Game-based learning is a type of active learning that uses the core elements, structures, and rules of games to lead learning experiences. In game-based learning, students are introduced to new concepts and skills and can practice and implement them in a low-risk setting. This is advantageous because students' retention and application of material is directly related to their development of understanding through game play. Games are a useful tool for teaching basic introductory library skills because skills required to win games align with core information literacy skills including problem-solving, planning, and critical thinking [1]. Skills associated with play include teamwork, communication, and delegation as well as critical thinking, attention to detail, and lateral thinking [2]. All of these skills are essential for students to develop and can be cultivated through escape rooms.

Escape rooms are “live-action team-based games where players discover clues, solve puzzles, and accomplish tasks in one or more rooms in order to accomplish a specific goal (usually escaping from the room) in a limited amount of time” [2]. Educational escape rooms operate in generally the same manner but are developed for a specific audience with well-defined learning objectives. From an instructional standpoint, escape room games are collaborative, task-centered, time-based, and provide immediate feedback, making them an attractive consideration for in-class learning activities [3].

Escape rooms have become a global phenomenon since the earliest known ‘real’ escape room opened in Kyoto, Japan in 2007 [4]. There are now estimated to be over 50,000 escape rooms worldwide [5] and the industry has grown rapidly in the United States. While in 2014 there were only about two dozen escape room facilities in the United States, there are currently over 2,000 [6-7]. Parallel to the increasing popularity of recreational escape rooms globally, escape rooms are increasingly being used in higher education as an adjunct to traditional teaching methods [8]. The implementation of educational escape rooms in higher education has shown great value in learner outcomes, perceptions, and engagement [9-14]. Educational escape rooms can contribute to student engagement, cooperation, and content mastery and enhance an in-person classroom format [2, 15-16].

Recent reviews have shown that the majority of educational escape rooms cover subjects related to various medical and health sciences disciplines, followed closely by educational escape rooms developed for science, technology, engineering, and mathematics (STEM) education [16-17]. Simultaneously, academic libraries are increasingly using escape rooms for various educational purposes including library orientation and introducing students to institutional services [18-23], observing students' information search behavior [24], and teaching concepts in information literacy [25]. Literature on the use of escape rooms to teach library skills is still limited, especially in health sciences libraries [26-27]. However, the “rise” of escape rooms in health professions education generally [28] offers librarians an opportunity to incorporate this method into their instruction.

The escape rooms described in this paper offered unique experiences for students through active learning. HSL initially piloted an escape room activity in an in-person team-based optometry course in spring 2019. The pharmacy librarian was subsequently approached in January 2020 by pharmacy faculty about modifying the escape room activity to meet the needs of pharmacy students. An escape room activity also took place during the online orientation of the inaugural medical class in July 2020. The development and execution of these activities is described in the Case Presentation below.

## CASE PRESENTATION

In developing escape room activities for optometry, pharmacy, and medical students, the prompt for each escape room scenario was formatted as a research proposal. The rationale was to connect the development of library skills to a real-world, practical, and beneficial outcome. The following is an example of a prompt used with optometry students:

**You have been assigned a group project to investigate the feasibility of developing a new vision screening program for underserved children in Houston. You need to find journal articles to help flesh out the details of the project idea and provide supporting evidence for an upcoming grant application.**

**You thought it would be easy! You thought you could just find everything you need on Google… until Dr. Richdale told you that you'll need to use library resources.**

**Now everyone's worst nightmare is real: you're stuck at the library and can't leave until you find a relevant article. You're going to need to get to work…**

**Your team will have 45 minutes to navigate through library resources and accomplish this task.**

In order to emulate entertainment escape rooms, the final task in each activity was to find a four-digit number that was then used to unlock either a physical or a virtual combination lock. In some iterations, this number was a particular journal's impact factor, and in others it was the starting page number of an article included in a relevant search result's reference list.

### In-Person Team-Based Escape Room

In 2019, an in-person escape room was designed for optometry students using existing space and personnel, and minimal supplies.

Community Health is a required 2-credit hour course for approximately 100 first-year Doctor of Optometry students. The course is designed to acquaint students with the organization of the health care delivery system and to provide the underpinnings of the profession including its history and socioeconomic, ethical, and legal elements. It includes an introduction to epidemiology, biostatistics, and evidence-based practice.

The purpose of this escape room activity was to add an active learning component to the standard library lecture on information literacy concepts. This took place over two one-hour sessions. In the first, students attended an in-class lecture which prepared them with the necessary background information to enable successful completion of the game. In the second session, students participated in the escape room activity. This consisted of working in teams of five to six students to complete a variety of tasks, including both paper and web-based puzzles, that tested knowledge and skills related to obtaining supporting literature for an evidence-based practice research proposal. Each team was allowed to use one computer; other electronic devices such as phones and tablets were prohibited. Teams were also required to explore and move throughout the library's physical space to locate library materials. The web-based puzzles were created in Google Forms and Google Docs. Solving them required the use of the library website and databases including PubMed and Journal Citation Reports. Two librarians and one optometry faculty member served as game facilitators. Teams were considered to have successfully 'escaped' when they were able to correctly complete all game tasks in sequence and obtain the code to unlock a lock box within 45 minutes. Teams could ask for hints if they were stuck, but were given a one-minute penalty for each hint. Teams signed out on paper and recorded their finishing time in front of a facilitator. As part of the course, points were assigned based on completion time.

The activity was repeated in 2020 with improvements based on feedback from the first cohort. In order to avoid crowding at the physical location of clues, two tracks were created. The clues in each track were of similar content and level of difficulty, but led the teams to two different sites. Perhaps due to the reduced physical crowding, all teams were able to 'escape' faster than the previous group.

### On-Site Virtual Individual Escape Room

An individual escape room activity utilizing similar resources was piloted with pharmacy students in 2020.

Literature Evaluation is a required 2-credit hour course taught to approximately 130 second-year students in the Doctor of Pharmacy program. The course is designed to instill essential skills to help pharmacy students analyze, interpret, and critically evaluate medical literature and answer patient care or drug-related questions.

Similar to the optometry escape room, the purpose of this escape room activity was to assess the students' learning from their lecture on literature evaluation provided earlier in the semester by a member of the pharmacy faculty. Based on the content of this lecture, the pharmacy librarian created a series of exclusively web-based clues using Google Forms and Google Docs. While there was some content overlap with the previously-created optometry activity, the focus of the lectures was different, so few of the clues could be reused. New content covered included using the MeSH Database and PubMed Search Builder.

The activity took place in a large lecture hall. Each student was seated individually with their own laptop. The pharmacy librarian provided instructions and the prompt, and then students completed the activity on their own. If a student could not move forward, they could raise their hand and a facilitator would give them individual help. One librarian, two pharmacy faculty members, and several graduate assistants served as game facilitators. At certain points throughout the allotted time, clues were provided to the room at large to ensure students could complete the activity in time. Each student was considered to have successfully 'escaped' when they were able to correctly complete all game tasks in sequence and obtain the code to unlock a lock box within 45 minutes. In order to keep a game-like quality to the activity despite it being completed online individually, physical lock boxes filled with candy were used for the final task. Upon completion, students signed out and left the room. Grades were assigned based on completion/non-completion of the activity.

### Fully Remote Virtual Individual Escape Room

Also in 2020, an escape room was developed as part of the first-year Doctor of Medicine students' orientation. Due to the COVID-19 pandemic, this activity was conducted fully online. The 90-minute session was designed to familiarize the thirty medical students with library resources and the medical librarian, as well as provide a brief introduction to information literacy concepts. Again, the librarian created a series of exclusively web-based clues using Google Forms and Google Docs. While there was some content overlap with the previous activities, this iteration was not tied to a specific course and as such focused somewhat less on information literacy and research skills and more on ensuring the students were able to access library resources.

The online session began with a 35-minute lecture and demonstration of the library website, database/full-text access, information literacy concepts, and PubMed searching. The students were then given the prompt and completed the activity independently. If a student could not move forward, they sent a direct message to the librarian through the meeting platform to get help. The medical librarian acted as the sole facilitator. At certain points throughout the allotted time, clues were provided to the group at large to ensure students completed the activity in time. Each student was considered to have successfully 'escaped' when they were able to correctly complete all game tasks in sequence and obtain the code to unlock a virtual box within 45 minutes. There was a short debriefing session at the end of the session. Grades were not assigned for the activity.

The 2021 optometry student escape room also took place fully online and incorporated the lessons learned from the pharmacy and medical student activities. Table 1 provides a complete listing of the escape rooms described in this report, including date, health profession, format, and learning objectives. The supplemental file contains a complete example of a virtual individual escape room activity for optometry.

**Table 1:** Library Escape Rooms for Health Professions Students

Date	Health Profession	Setting/Format	Learning Objectives
March 2019 & January 2020	Optometry	In-Person / Team	Locate Optometry Resources guide Identify an empirical research article Describe peer review Find journal impact factor Apply PICO to database search Use PubMed to obtain full-text articles Demonstrate ILLiad account setup Know how & who to ask for help
March 2020	Pharmacy	Hybrid / Individual	Locate Pharmacy Resources guide Use MeSH Database to identify appropriate MeSH terms Use PubMed Search Builder to construct search utilizing MeSH terms in PubMed Use PubMed to obtain full-text articles
July 2020	Medicine	Online / Individual	Locate Medicine Resources guide Apply PICO to database search Use PubMed to obtain full-text articles Demonstrate ILLiad account setup Know how & who to ask for help
February 2021	Optometry	Online / Individual	Locate Optometry Resources guide Identify an empirical research article Describe peer review Find journal impact factor Apply PICO to database search Apply MEDLINE filter to PubMed search Use PubMed to obtain full-text articles Know how & who to ask for help

## DISCUSSION

The activities described in this case report show that escape rooms can be successful in a variety of formats for a variety of learners. The formative assessments embedded into the activities demonstrated that learning objectives were met: students were able to successfully complete all game tasks in the allotted time. Evaluation of the activities was informal. Observations of and interactions with the students while participating in the escape rooms revealed an overall high level of enjoyment and engagement. Anecdotal feedback showed that the activities were well-received by the participating students in optometry, pharmacy, and medicine. Additionally, the flexibility of this activity allowed librarians and instructors to adapt to the changing public health landscape.

There are several important decisions to be made when incorporating an escape room into health sciences library instruction. One is whether the activity should be planned as a team-based or individual activity format. Team-based activities can encourage development of communication and collaboration skills. Individual activities ensure that each student demonstrates individual proficiency, rather than relying on teammates. Either format can be successful depending on the individual program goals.

Another important consideration is cost, in both time and money. The considerable time that can be spent on planning decreases with each iteration. Planning the first escape room could take approximately 12 hours, with each additional activity requiring three to five hours to plan. The decrease in time spent for subsequent events holds even when adjusting audiences and/or formats. A small budget may be needed for supplies and/or prizes, but it is often possible to use items from the library's general supplies cache without spending extra money. Virtual clues and escape rooms can utilize freely-available online tools such as web forms, documents, and QR codes. In fact, a fully online activity may not incur additional costs.

Related to the consideration of cost is setting. In-person escape rooms may feel more active and fun to students while allowing them to become familiar with the physical library space, but will require more resources than virtual activities. Additionally, since health sciences information literacy skills are typically practiced in an online environment, virtual settings are well-suited for this learning. At times, online activities may be the only option due to public health concerns.

There are also benefits and drawbacks to consider when deciding whether to provide a score/grade for the escape room. Attaching points to the activity may reinforce the idea that it is an important part of the course and that full participation is expected. However, students may learn better in a risk-free environment where activities are not tied to grades. Feedback from the optometry students who received points based on their team rankings suggested that some groups were concerned with the “fairness” of points based on hints given or the potential for cheating by other students and thus may have focused more on their grade than their newly-learned skills. The pharmacy students only received completion points and in-room facilitators helped when students were stuck, ensuring all students completed the activity and earned the same number of points. The goal was that all students were able to escape at their own pace without creating a race or grade-based environment. Most students finished early, allowing the facilitators to spend more time with those having trouble. Instructors should determine which scenario fits best with their learners and courses.

Planners and facilitators should be aware of possible barriers for learners for whom English is not their first language. For example, difficulties with spelling and grammar may lead to errors and require additional time and tries to obtain the answers. Additionally, clues that are dependent on familiarity with English idioms, puns, or expressions should be avoided. Team-based formats may be beneficial for these students, such that they can contribute and practice their library skills without being responsible for all aspects of searching and writing.

Important lessons learned from planning these activities include that it is advisable to start with learning objectives then work backwards [29] to develop clues and questions. When designing the flow of the activity, start with a simple clue to build the students' confidence. It is necessary to have colleagues run through the activity to check for errors and potential alternate clue interpretations prior to implementation. Finally, there is typically a wide range of time required for students to escape. Plan for the early finishers to have some free time while the rest of the class completes the activity.

Escape rooms can be an effective strategy for library instruction in the health sciences. They can work in multiple formats to bring game-based learning to a variety of health professions students.

## Data Availability

There are no data associated with this article.

## SUPPLEMENTAL FILES

**NA**
